# Intramedullary Nailing Versus Plate Fixation for AO/OTA Types 43A and 43B Distal Tibial Fractures: Early Pain Trajectories, 3-Month Ankle Function, and Return to Work in a Retrospective Cohort

**DOI:** 10.3390/jcm15176752

**Published:** 2026-08-31

**Authors:** Feng-Jun Lan, Chang-Sheng Liao, Huan-Sheng Liu, Dai-Xin Tan, Cheng Long

**Affiliations:** 1 Department of Orthopaedics, West China Hospital, Sichuan University, Chengdu 610041, China; 17340206341@163.com (F.-J.L.); 15603441409@163.com (C.-S.L.); lhs200006@163.com (H.-S.L.); tandaixin0708@163.com (D.-X.T.); 2 Trauma Medical Center, Department of Orthopaedic Surgery, West China Hospital, Sichuan University, Chengdu 610041, China

**Keywords:** distal tibial fracture, intramedullary nailing, plate fixation, pain trajectory, return to work, AOFAS, overlap weighting

## Abstract

**Background/Objectives**: Intramedullary nailing (IMN) and plate fixation are established treatments for distal tibial fractures, but differences in early pain, occupational recovery, and short-term function remain uncertain in routine practice. This study examined associations between fixation strategy and early recovery profiles and described union-related complications, fixation failure, and reoperation in AO/OTA 43A-B distal tibial fractures. **Methods**: This single-center retrospective comparative cohort included 87 adults treated between January 2019 and December 2025 (IMN, *n* = 35; plate fixation, *n* = 52). The primary outcome was the 3-month American Orthopaedic Foot and Ankle Society (AOFAS) ankle-hindfoot score. Secondary outcomes included serial visual analog scale (VAS) pain scores through 2 years, return-to-work time, perioperative outcomes, deep-vein thrombosis, infection, delayed union, nonunion, internal-fixation failure, and reoperation. Multivariable regression, generalized estimating equations, propensity-score overlap weighting, and a sensitivity model additionally accounting for associated fibular fracture and fixation were used. **Results**: After multivariable adjustment, IMN was associated with a 2.53-point higher 3-month AOFAS score (95% confidence interval [CI], 0.48–4.57; *p* = 0.015) and return to work 30.8 days earlier (95% CI, 20.0–41.5 days earlier; *p* < 0.001). Adjusted VAS scores favored IMN on postoperative day 1 (−1.85), week 1 (−1.31), and month 1 (−1.88), with a significant treatment-by-time interaction (*p* < 0.001). By 6 months, 1 year, and 2 years, mean pain scores were near zero in both groups. Overlap-weighted estimates and analyses additionally accounting for associated fibular fracture and fibular fixation remained directionally consistent. Delayed union, nonunion, fixation failure, and reoperation were uncommon and did not differ statistically between groups, although event counts were small. **Conclusions**: In appropriately selected AO/OTA 43A-B fractures, IMN was associated with less early postoperative pain, earlier occupational recovery, and a modestly higher 3-month AOFAS score. These observational findings characterize early recovery rather than establish universal implant superiority; low event counts preclude definitive conclusions regarding structural complications.

## 1. Introduction

Distal tibial fractures are difficult to manage because the distal tibia has limited soft-tissue coverage, a vulnerable blood supply, and a short metaphyseal segment adjacent to the ankle joint. AO/OTA 43A fractures are extra-articular, whereas 43B fractures involve part of the articular surface [1]. Successful fixation must restore length, rotation, and alignment while limiting additional injury to the soft-tissue envelope [2,3].

Intramedullary nailing (IMN) is a load-sharing technique that usually requires less exposure of the distal tibia and may facilitate early mobilization. Modern multiplanar distal interlocking options have expanded its use, although a short distal fragment, metaphyseal widening, malalignment, and anterior knee pain remain concerns [2,3,4,5,6,7,8]. Plate fixation offers direct or indirect reduction and strong control of the distal segment, but wound problems, implant prominence, and soft-tissue irritation may occur, especially when the medial soft-tissue envelope is compromised [3,4,5,6,7,8,9].

Randomized trials have not demonstrated universal superiority of either method. The UK FixDT trial found similar disability at 6 months but faster recovery during the early postoperative period after IMN, and the nail strategy was more likely to be cost-effective [10,11,12]. Meta-analyses suggest that IMN may reduce wound complications and accelerate weight bearing or union, whereas plate fixation may improve control of alignment; pooled functional scores are generally similar [13,14,15,16]. Five-year FixDT follow-up further indicates that most recovery occurs during the first year and that residual disability can persist regardless of fixation method [17].

These findings shift the clinically relevant question from whether one implant is universally superior to how fixation strategy is associated with early recovery under real-world clinical selection. The present study therefore evaluates whether fixation strategy was associated with differences in serial postoperative pain, 3-month ankle function, return to work, perioperative burden, and short-term complications among adults with AO/OTA 43A-B distal tibial fractures. We hypothesized that IMN would be associated with lower early postoperative pain, a higher 3-month AOFAS score, and earlier return to work.

## 2. Materials and Methods

### 2.1. Study Design, Ethics, and Data Source

This single-center retrospective comparative cohort study was conducted in the Department of Orthopaedics, West China Hospital, Sichuan University, Chengdu, China. Consecutive eligible patients treated between January 2019 and December 2025 were identified from the institutional clinical database. Clinical, surgical, and follow-up variables were generated during routine diagnosis, treatment, and postoperative care. No research-specific intervention or additional follow-up was performed. Formal data extraction, de-identification, and statistical analysis were conducted after ethics approval.

The study was conducted in accordance with the Declaration of Helsinki and approved by the Biomedical Ethics Review Committee of West China Hospital, Sichuan University (protocol code 2026 Review (1252) approved on 26 March 2026). The requirement for written informed consent was waived because of the retrospective design and use of de-identified routinely collected data. Reporting followed the STROBE statement [18].

### 2.2. Participants

Patients were eligible if they were aged 18 years or older; had a unilateral AO/OTA 43A or 43B distal tibial fracture; underwent definitive fixation with a standard antegrade locked intramedullary nail or plate fixation; underwent surgery within 14 days after injury; and had at least 6 months of routine follow-up data. Exclusion criteria were open or pathological fracture, major neurovascular injury, bilateral injury, previous ipsilateral tibial or ankle fracture, severe systemic disease precluding the selected operation, or missing primary-outcome data. Complete articular AO/OTA 43C fractures were excluded.

The predefined database query identified 89 unique patient records. Two patients younger than 18 years were excluded in accordance with the adult eligibility criterion, leaving 87 adults for the final analysis (IMN, *n* = 35; plate fixation, *n* = 52). No additional patient met any of the other predefined exclusion criteria, including open or pathological fracture, major neurovascular injury, bilateral injury, previous ipsilateral tibial or ankle fracture, severe systemic disease precluding the selected operation, insufficient minimum follow-up, or missing 3-month AOFAS data. No eligible patient was lost before the minimum 6-month follow-up. Associated fibular fracture/fixation and structural follow-up outcomes were abstracted from pre-existing routine clinical follow-up records generated during patient care; no prospective research-specific follow-up or outcome collection was performed.

### 2.3. Treatment Strategies

Treatment allocation was not randomized. The operative strategy was selected by the attending trauma team according to fracture morphology, articular extension, the length and stability of the distal segment, soft-tissue condition, associated fibular injury, patient characteristics, and surgeon judgment. In the IMN group, closed or limited-open reduction was followed by locked antegrade tibial nail fixation with proximal and distal interlocking screws. Blocking screws and temporary reduction aids were used when needed to maintain alignment. In AO/OTA 43B fractures, the partial articular component was reduced and stabilized before or in conjunction with definitive fixation, including independent lag-screw fixation when indicated by fracture morphology. Because these clinical factors also influenced treatment selection, between-group comparisons were interpreted as adjusted observational associations rather than causal treatment effects.

In the plate-fixation group, open reduction or minimally invasive plate osteosynthesis was selected according to fracture pattern and soft-tissue conditions. Distal tibial locking plates were used to obtain stable fixation and restore alignment. Associated fibular fractures were fixed when this was considered necessary to restore length, rotation, ankle stability, or facilitate tibial reduction.

### 2.4. Perioperative Management and Rehabilitation

Postoperative management followed institutional clinical practice. All patients received perioperative antibiotic prophylaxis, multimodal analgesia, wound care, and venous thromboembolism prophylaxis unless contraindicated. Ankle-pump exercises, quadriceps isometric contraction, and ankle range-of-motion exercises were encouraged as tolerated. Progression from protected weight bearing to partial and then full weight bearing was individualized according to soft-tissue recovery, fixation stability, symptoms, and radiographic evidence of healing.

### 2.5. Outcomes

The primary outcome was the AOFAS ankle–hindfoot score at 3 months. The scale ranges from 0 to 100, with higher scores indicating better pain, function, and alignment. Although widely used in ankle trauma, it combines clinician- and patient-reported domains and may demonstrate ceiling effects [19,20].

Secondary outcomes were the interval from admission to definitive fixation; postoperative hospital stay; operative time; intraoperative blood loss; time from surgery to documented return to preinjury or modified occupational duties; and VAS pain scores preoperatively and at postoperative day 1, week 1, month 1, month 6, year 1, and year 2. All 87 included patients were occupationally active before injury; none were retired or unemployed at baseline, and return-to-work dates were available for all included patients. Occupation category, physical workload, compensation status, and employer accommodations were not systematically recorded. The admission-to-fixation interval was interpreted as a treatment-pathway measure rather than a biological effect of the implant.

Lower-extremity venous duplex ultrasonography was performed during perioperative care according to institutional screening practice and repeated when clinical findings or laboratory abnormalities raised concern for thrombosis. Deep-vein thrombosis (DVT) was defined by venous non-compressibility or direct visualization of an intraluminal thrombus on ultrasonography and was categorized as perioperative DVT regardless of symptom status. Postoperative infection was defined as a documented surgical-site infection requiring antimicrobial treatment and/or surgical management during follow-up. Existing routine clinical follow-up records were also reviewed for delayed union, nonunion, internal-fixation failure, and reoperation. The timing of formal radiographic union was not standardized sufficiently in routine follow-up to support a continuous time-to-union comparison; therefore, we report clinically documented delayed union and nonunion rather than a time-to-union endpoint. Malunion and standardized coronal/sagittal alignment measurements were not consistently available and therefore were not analyzed as comparative endpoints.

### 2.6. Statistical Analysis

Continuous variables are presented as mean ± standard deviation and categorical variables as number and percentage. Welch’s *t* test was used for unadjusted continuous comparisons. Categorical comparisons used the chi-square test or Fisher’s exact test as appropriate. Standardized mean differences (SMDs) were calculated to describe baseline imbalance; absolute SMD < 0.10 was considered satisfactory balance after weighting. Because low-frequency structural complications generated sparse cells, delayed union, nonunion, fixation failure, and reoperation were compared descriptively with Fisher’s exact test and were not entered into multivariable complication models.

Multivariable linear regression estimated the adjusted mean difference for IMN relative to plate fixation. Models included age, sex, body mass index (BMI), AO/OTA type B versus type A fracture, smoking, alcohol use, diabetes, and hypertension, with HC3 heteroscedasticity-consistent standard errors. These covariates were selected a priori as clinically plausible confounders and to preserve model parsimony given the cohort size. Because associated fibular fracture and fibular fixation were clinically imbalanced between groups, an additional sensitivity analysis added both variables to the primary AOFAS and return-to-work models. This extended model was treated as sensitivity analysis rather than the primary model because fibular fixation is closely linked to fracture morphology and operative strategy.

Repeated early pain measurements from postoperative day 1 to month 1 were analyzed using generalized estimating equations with a Gaussian family and exchangeable working correlation [21]. The model included treatment group, time, treatment-by-time interaction, preoperative VAS, age, sex, BMI, fracture type, smoking, diabetes, and hypertension, with robust standard errors. Time-specific group contrasts were adjusted using the Holm method. A further sensitivity GEE added associated fibular fracture and fibular fixation.

As a separate sensitivity analysis, the propensity for IMN treatment was estimated from age, sex, BMI, fracture side, AO/OTA type, smoking, alcohol use, diabetes, and hypertension. Overlap weights were assigned as 1—propensity score for IMN patients and as the propensity score for plate-fixation patients [22]. Covariate balance before and after weighting was assessed with absolute SMDs. Weighted between-group associations were estimated with robust standard errors. Associated fibular fracture and fibular fixation were displayed in the baseline table but were not incorporated into the propensity model because fibular fixation forms part of the treatment pathway and was strongly associated with definitive fixation selection.

No a priori sample-size calculation was performed because all eligible patients identified in the predefined study period were included. Except for Holm-adjusted time-specific VAS contrasts, all tests were two-sided with *p* < 0.05 considered statistically significant. Low-frequency complication analyses were interpreted primarily through event counts and confidence in the direction of evidence rather than the absence of statistical significance. Analyses were performed in Python 3.13.5, statsmodels 0.14.6, and SciPy 1.17.0.

## 3. Results

### 3.1. Cohort Formation and Baseline Characteristics

**Figure 1:**
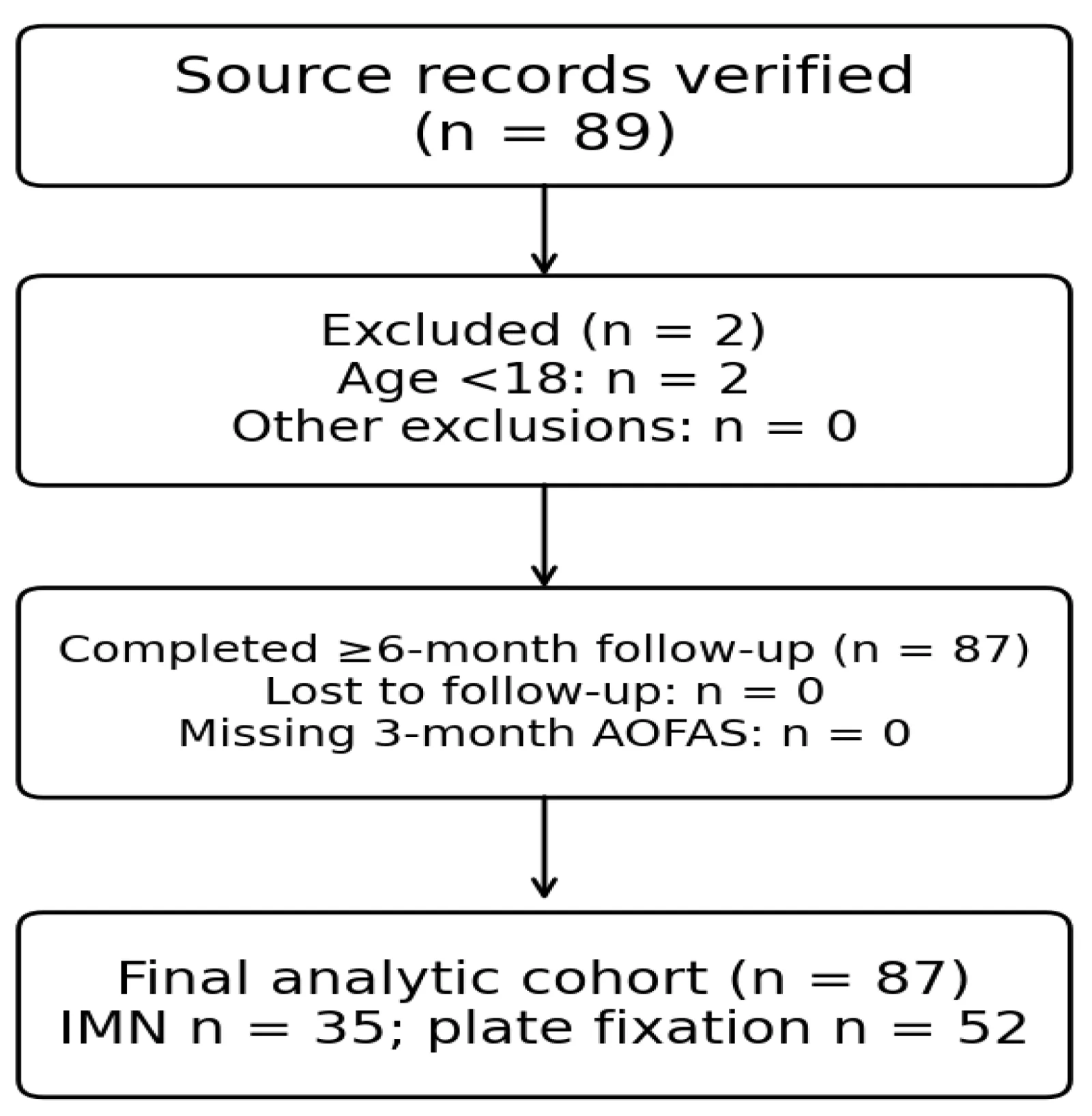
Study flow diagram. The predefined database query identified 89 unique patient records. Two patients aged <18 years were excluded; no other predefined exclusion criterion was met. Eighty-seven adults completed the minimum 6-month follow-up with no missing 3-month AOFAS outcome and were included in the analysis (IMN, *n* = 35; plate fixation, *n* = 52).

The predefined database query identified 89 unique patient records. Two patients aged <18 years were excluded according to the predefined adult eligibility criterion; no other predefined exclusion criterion was met. The final cohort therefore comprised 87 adults: 35 underwent IMN and 52 underwent plate fixation (Figure 1). No eligible patient was lost before the minimum 6-month follow-up. All 87 had the 3-month AOFAS outcome and the specified return-to-work and VAS records, and all were occupationally active before injury. Baseline age and most comorbidities were similar, but the IMN group had lower BMI and a higher proportion of men. Associated fibular fracture (71.4% vs. 92.3%) and fibular fixation (51.4% vs. 84.6%) were also less frequent in the IMN group, demonstrating clinically important treatment-selection heterogeneity (Table 1). These imbalances support cautious interpretation of the between-group estimates as adjusted observational associations.

**Figure 2:**
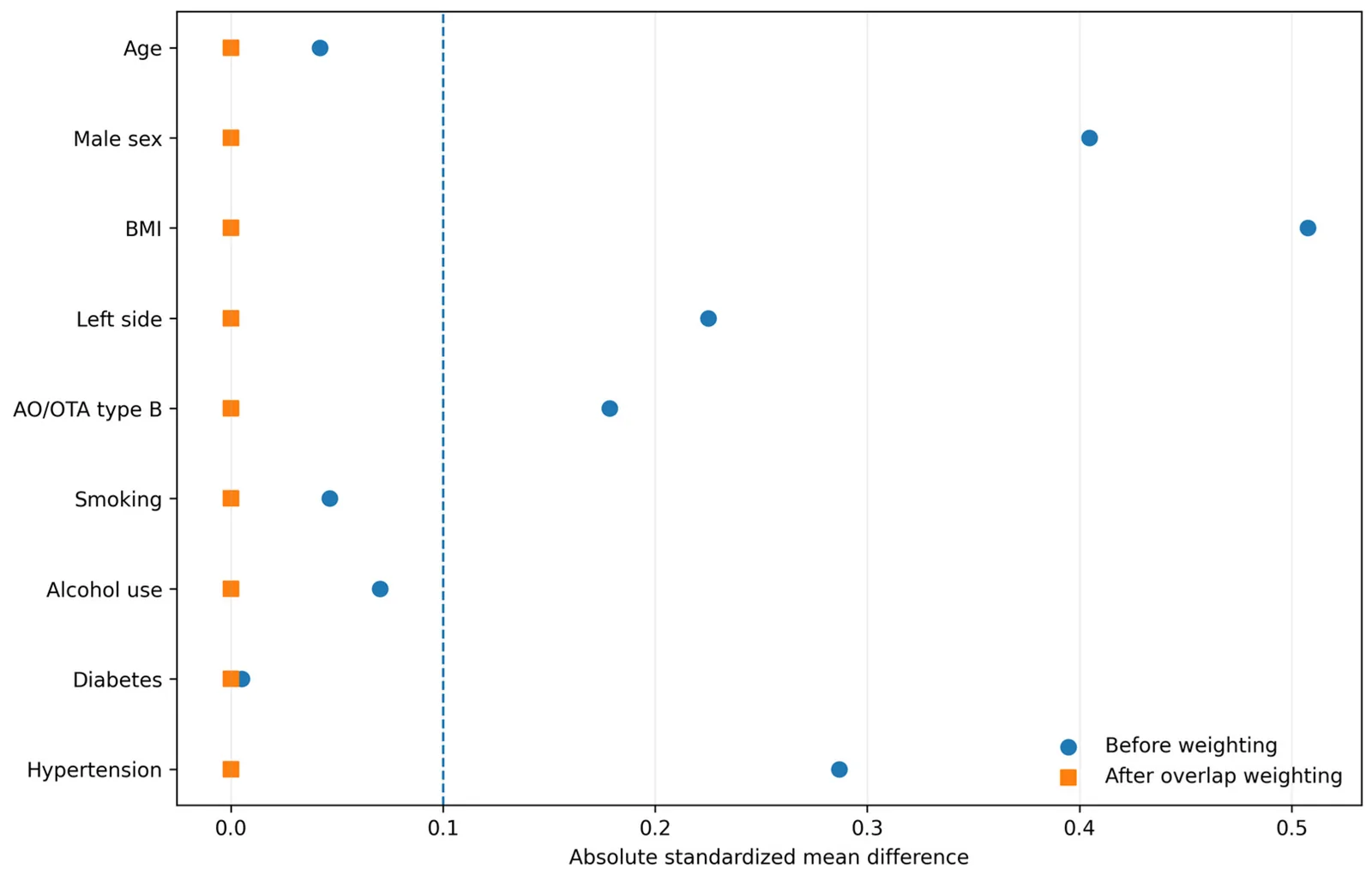
Absolute standardized mean differences before and after propensity-score overlap weighting. The dashed vertical line indicates the conventional balance threshold of 0.10. Weighting was based on age, sex, BMI, fracture side, AO/OTA type, smoking, alcohol use, diabetes, and hypertension. Associated fibular fracture/fixation are shown in Table 1 and addressed separately in sensitivity analysis.

Propensity scores showed usable but imperfect treatment overlap. Overlap weighting reduced the absolute SMDs of all covariates included in the propensity model to approximately zero (Figure 2). The marked unweighted imbalance in sex and BMI, together with differences in associated fibular injury and fixation, reinforces that the two treatment groups were not exchangeable at baseline and that residual confounding may remain despite adjustment.

### 3.2. Perioperative Outcomes, Return to Work, and Function

**Figure 3:**
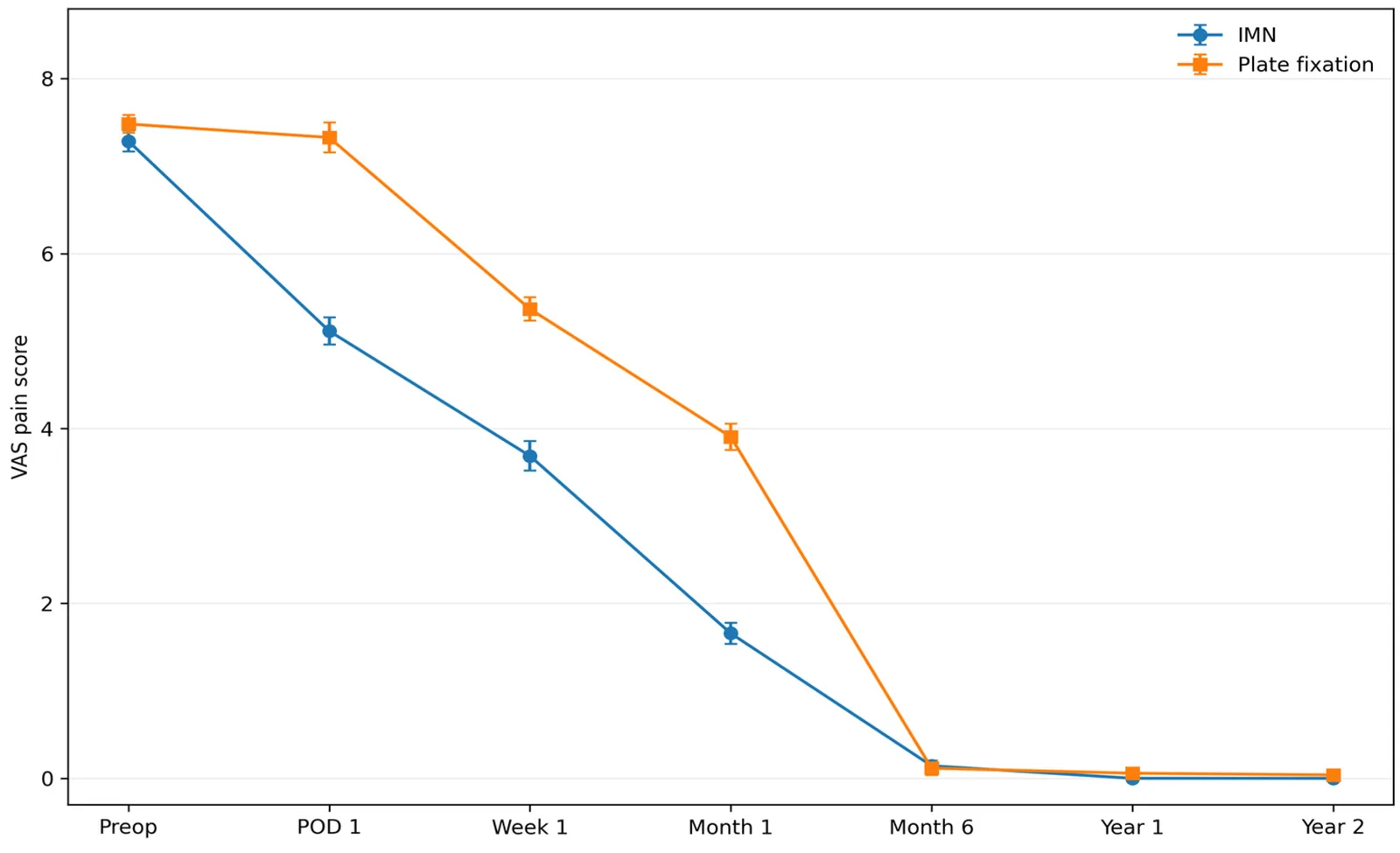
Visual analog scale (VAS) pain trajectory from the preoperative assessment through 2 years after surgery. Points show group means and error bars show standard errors. Adjusted treatment-by-time interaction for the early postoperative period (day 1 to month 1) was significant (*p* < 0.001). By 6 months and thereafter, mean VAS values were near zero in both groups.

Unadjusted analyses showed shorter admission-to-fixation intervals, less intraoperative blood loss, earlier return to work, and higher 3-month AOFAS scores in the IMN group. Early postoperative VAS scores were also lower after IMN. Longitudinal VAS records showed that pain had largely converged by 6 months and remained near the floor of the scale at 1 and 2 years in both groups (Table 2, Figure 3).

After multivariable adjustment, the 3-month AOFAS score was 2.53 points higher after IMN (95% CI, 0.48–4.57; *p* = 0.015). Return to work occurred 30.8 days earlier (95% CI, 20.0–41.5 days earlier; *p* < 0.001), and the admission-to-fixation interval was 1.94 days shorter (95% CI, 1.02–2.86 days shorter; *p* < 0.001). Adjusted operative time and postoperative hospital stay did not differ significantly. Intraoperative blood loss was numerically lower after IMN, but the adjusted difference did not meet the prespecified *p* < 0.05 threshold (−11.67 mL; 95% CI, −23.58 to 0.25; *p* = 0.055) (Table 3).

Overlap-weighted sensitivity analyses produced similar estimates for the AOFAS score (2.70 points higher; 95% CI, 0.52–4.87; *p* = 0.015), return-to-work time (30.6 days earlier; 95% CI, 17.3–44.0 days earlier; *p* < 0.001), and the admission-to-fixation interval (1.84 days shorter; 95% CI, 0.88–2.79 days shorter; *p* < 0.001). The weighted blood-loss estimate remained directionally favorable but nonsignificant (*p* = 0.063). In an additional sensitivity model accounting for associated fibular fracture and fibular fixation, the AOFAS association was attenuated but remained at the conventional significance threshold (+2.06 points; 95% CI, 0.04–4.08; *p* = 0.046), while the return-to-work association remained substantial (−29.97 days; 95% CI, −40.77 to −19.18; *p* < 0.001) (Supplementary Table S1).

### 3.3. Pain Trajectory

Preoperative VAS scores were similar between groups. Postoperative pain was lower in the IMN group at day 1, week 1, and month 1. The generalized estimating equation model demonstrated a significant treatment-by-time interaction from day 1 to month 1 (Wald *p* < 0.001), indicating different early pain trajectories. Figure 3 extends the descriptive trajectory through 2 years and shows convergence to near-zero scores after the early postoperative period.

The adjusted mean VAS difference favored IMN by −1.85 points on day 1 (95% CI, −2.34 to −1.35), −1.31 points at week 1 (95% CI, −1.79 to −0.83), and −1.88 points at month 1 (95% CI, −2.32 to −1.44); all Holm-adjusted *p* values were <0.001 (Table 4). Additional adjustment for associated fibular fracture and fibular fixation yielded similar early differences (−1.81, −1.28, and −1.85 points, respectively). At 6 months, mean VAS scores were 0.14 ± 0.36 after IMN and 0.12 ± 0.58 after plate fixation (*p* = 0.786); at 1 and 2 years, both groups remained near the floor of the scale, indicating that the observed pain difference was primarily an early-recovery phenomenon.

### 3.4. Fracture-Healing and Complication Outcomes

Perioperative DVT occurred in 10 of 35 patients (28.6%) after IMN and 12 of 52 patients (23.1%) after plate fixation (Fisher’s exact *p* = 0.620). Postoperative infection occurred in 0 of 35 and 3 of 52 patients (5.8%), respectively (*p* = 0.270). Delayed union occurred in 1 of 35 (2.9%) versus 3 of 52 (5.8%; *p* = 0.646), nonunion in 0 versus 1 (1.9%; *p* = 1.000), internal-fixation failure in 0 versus 3 (5.8%; *p* = 0.270), and reoperation in 0 versus 4 (7.7%; *p* = 0.145). These events were uncommon, and the study was not powered to establish equivalence or superiority for structural complications. Standardized malunion and alignment data were unavailable.

### 3.5. Representative Radiographic Cases

**Figure 4:**
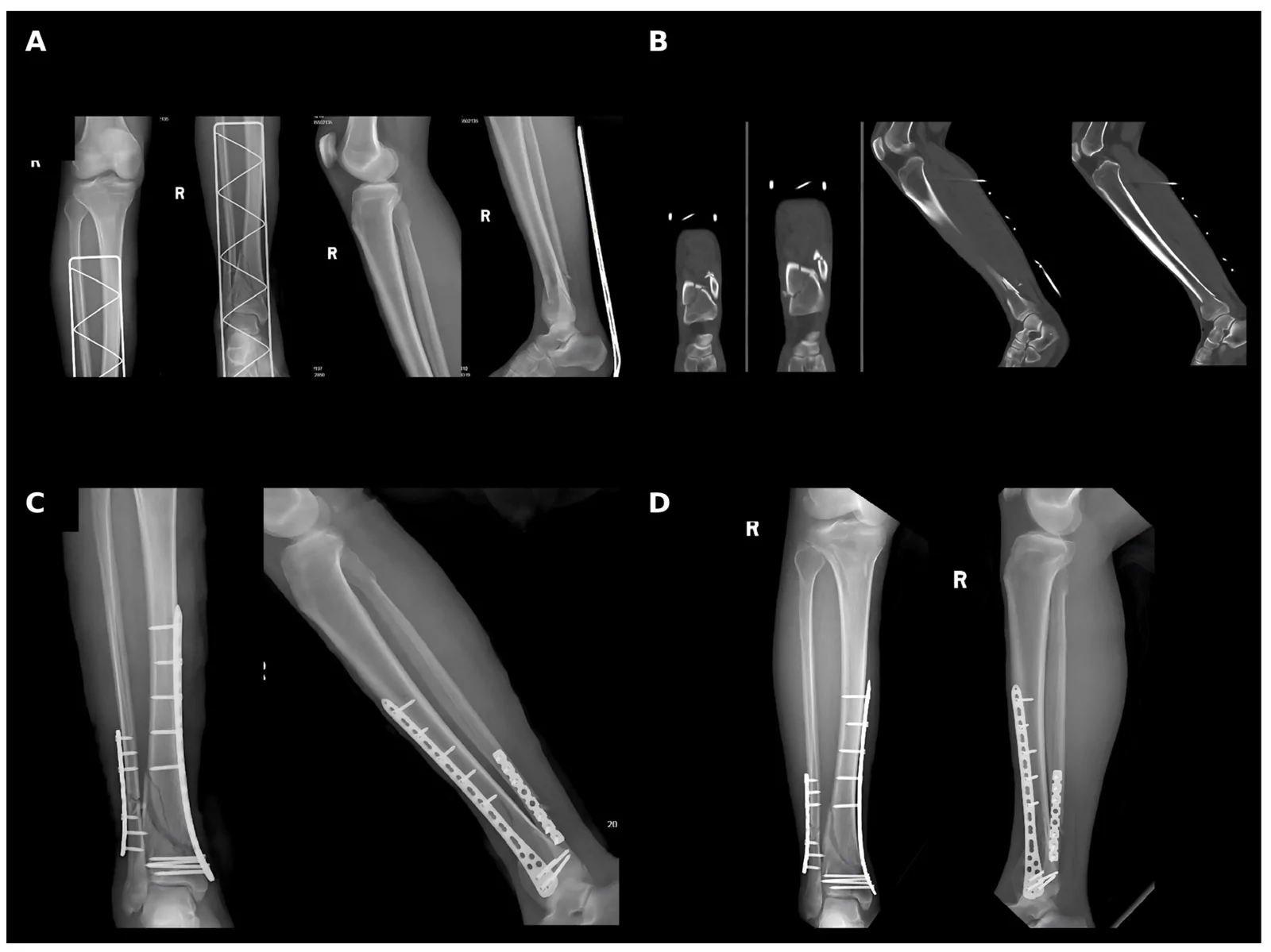
Representative case treated with plate fixation. (**A**) Preoperative anteroposterior and lateral radiographs showing a distal tibial fracture; (**B**) preoperative computed-tomography images demonstrating fracture morphology; (**C**) postoperative anteroposterior and lateral radiographs after distal tibial plate fixation with supplemental fibular fixation; (**D**) follow-up radiographs showing maintained implant position and alignment with interval fracture healing. R, right side.

**Figure 5:**
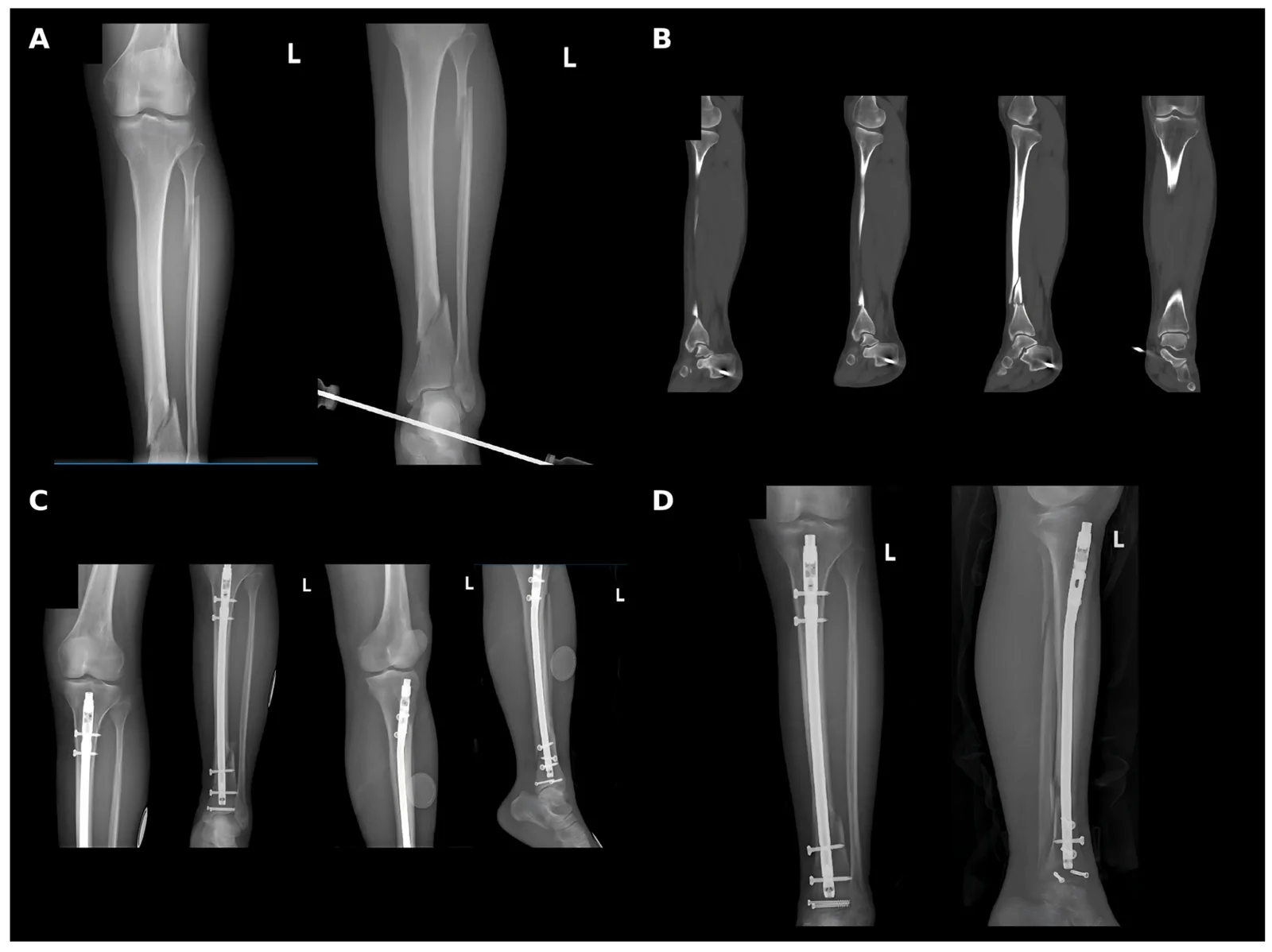
Representative case treated with intramedullary nailing. (**A**) Preoperative anteroposterior and lateral radiographs showing a distal tibial fracture; (**B**) preoperative computed-tomography images demonstrating fracture morphology; (**C**) postoperative anteroposterior and lateral radiographs after locked antegrade intramedullary nailing with distal interlocking; (**D**) follow-up radiographs showing maintained implant position and alignment with interval fracture healing. L, left side.

Representative radiographic sequences are shown in Figure 4 and Figure 5. The plate-fixation example illustrates preoperative radiographs and computed tomography, postoperative plate fixation of the distal tibia with supplemental fibular fixation, and maintained fixation at follow-up. The intramedullary-nailing example illustrates the preoperative fracture pattern, computed tomography, antegrade locked nailing with distal interlocking, and maintained alignment at follow-up. These images are provided for technical illustration and were not used as independent outcome adjudication.

## 4. Discussion

The principal contribution of this study is not another claim that one implant is universally superior, but a characterization of early recovery under real-world treatment selection. After multivariable adjustment and overlap weighting, IMN remained associated with less pain during the first postoperative month and earlier occupational recovery, while the 3-month AOFAS difference was statistically significant but modest. Structural outcomes were uncommon and did not show statistically demonstrable between-group differences; however, the event counts were too small for firm conclusions. The pronounced imbalance in associated fibular injury/fixation and the nonrandomized treatment pathway underscore that these findings should be interpreted as observational associations.

Our results complement rather than supersede the UK FixDT randomized trial. FixDT found similar 6-month disability between locking plates and intramedullary nails but faster early recovery after IMN [10,11], and its economic evaluation favored the nail strategy in the studied population [12]. Five-year follow-up showed that most recovery occurred within the first year and that residual disability could persist irrespective of fixation method [17]. The added value of the present cohort lies in three clinically specific dimensions: serial early postoperative pain trajectories analyzed longitudinally rather than as isolated pain time points; documented time to resumption of preinjury or modified occupational duties as a patient-centered recovery marker; and real-world inclusion of AO/OTA 43A fractures together with selected 43B partial-articular fractures, allowing early recovery to be examined within contemporary treatment-selection patterns. These features provide complementary observational evidence and do not establish a general hierarchy between implants. The consistency of the early-recovery signal with FixDT is reassuring, but the observational design cannot provide the same causal inference as randomization.

The early pain difference is clinically plausible. IMN generally avoids extensive exposure of the distal tibial surface, potentially limiting periosteal stripping, wound tension, and implant prominence. Nevertheless, pain is multifactorial and may also reflect fracture morphology, soft-tissue injury, adjunctive fibular procedures, analgesic practice, and rehabilitation. The approximately 1.3–1.9 point adjusted early differences are large enough to be perceptible in many postoperative settings, but no distal-tibia-specific minimal clinically important difference was prespecified. Importantly, the 6-month, 1-year, and 2-year VAS data show near-floor scores in both groups, indicating that the between-group pain signal should be framed as faster early pain resolution rather than durable long-term analgesic superiority. Other comparative clinical studies have also evaluated intramedullary nailing and minimally invasive plate osteosynthesis for distal tibial fractures [23]. However, direct comparison with the present pain trajectory is limited because previous studies have generally emphasized disability and global function rather than serial late VAS scores. In FixDT, substantial recovery occurred during the first year but residual disability could persist at longer follow-up [17]. Thus, near-zero late VAS values in our cohort should not be interpreted as complete fracture recovery or absence of residual functional limitation; they more likely represent a pain-specific floor effect within a coarse 0–10 scale.

Return to work occurred approximately one month earlier after IMN after adjustment and remained approximately 30 days earlier after additional control for associated fibular fracture/fixation. All included patients were occupationally active before injury; no retired or unemployed patient contributed to this endpoint. Nevertheless, return to work integrates pain control, mobility, confidence, rehabilitation, and functional recovery and is also influenced by occupation type, physical workload, compensation, employer accommodation, and socioeconomic context. Because these occupational characteristics were unavailable, the return-to-work difference should not be interpreted as a pure implant effect. The result is best viewed as a patient-centered marker of recovery within this institutional treatment pathway.

The adjusted AOFAS difference of 2.53 points was statistically significant but modest, and it attenuated to 2.06 points when associated fibular injury/fixation were added to the model. The AOFAS scale may have ceiling effects and combines clinician- and patient-reported domains [19,20]. Moreover, a 3-month assessment occurs before definitive union in many distal tibial fractures and should not be interpreted as a surrogate for fracture healing or durable comparative effectiveness. We therefore treat the 3-month AOFAS score as an early functional marker and interpret it together with the longer-term pain trajectory and the available delayed-union, nonunion, fixation-failure, infection, and reoperation data. Accordingly, the AOFAS result should not be used to argue for broad functional superiority.

### 4.1. Interpretation of Comparative Validity and Confounding by Indication

The central methodological limitation is that this cohort cannot establish comparative effectiveness in the causal sense. Treatment allocation was driven by fracture morphology, articular extension, distal-segment geometry, soft-tissue condition, associated fibular injury, and surgeon judgment, several of which were incompletely captured in the retrospective dataset. Consequently, the IMN and plate groups should not be regarded as exchangeable populations even after multivariable adjustment or overlap weighting. The analyses are therefore intended to estimate adjusted associations within clinically selected treatment pathways, not the effect that would be expected if otherwise identical fractures were randomized to the two implants.

The shorter admission-to-fixation interval observed in the IMN group should not be interpreted as a biological treatment effect. It likely reflects treatment pathway and selection: patients with greater soft-tissue concerns, more complex distal anatomy, or associated fibular injury may require staged optimization and may be preferentially treated with plates. This interpretation is consistent with the substantially higher prevalence of associated fibular fracture and fibular fixation in the plate group. This imbalance is clinically informative but also represents a source of confounding by indication.

Review of routine clinical follow-up records identified delayed union, nonunion, fixation failure, infection, and reoperation. These data were derived from records generated during clinical care without prospective research-specific follow-up. Delayed union, nonunion, fixation failure, and reoperation were numerically more frequent after plate fixation, but absolute event counts were low and none of these comparisons were statistically conclusive. The absence of a significant difference must not be interpreted as equivalence. Malunion, standardized coronal/sagittal alignment, and detailed hardware-removal indications were not consistently captured. These structural outcomes remain central to technique selection and require larger prospective cohorts with standardized radiographic follow-up.

Strengths include a clearly defined adult cohort, record-level cross-checking, repeated pain measurements extending to 2 years, a patient-centered return-to-work outcome, multivariable adjustment, robust standard errors, propensity-score overlap weighting, and an additional sensitivity analysis addressing the observed imbalance in associated fibular injury and fixation.

This study has important limitations. First, its retrospective, nonrandomized, single-center design and modest sample size create substantial potential for confounding by indication and residual selection bias. The two groups were not exchangeable at baseline: sex, BMI, associated fibular fracture, and fibular fixation differed meaningfully, and detailed fracture morphology, displacement, soft-tissue severity, surgeon preference, plate approach (open versus minimally invasive), and rehabilitation intensity were incompletely captured. Although overlap weighting balanced measured propensity-model covariates, and the fibular sensitivity model was directionally consistent, unmeasured confounding remains and the study cannot support a causal comparison of treatment modalities. Second, occupational category, workload, compensation, and employer accommodations were unavailable despite all participants being occupationally active before injury, limiting causal interpretation of return-to-work time. Third, delayed union, nonunion, fixation failure, and reoperation were rare, so the study was underpowered for structural complications; standardized time-to-radiographic union, malunion, and coronal/sagittal alignment measures were unavailable. Fourth, the 3-month AOFAS score is an early composite outcome and should not be interpreted as evidence of fracture union or durable superiority. Fifth, the near-zero VAS scores after 6 months create a floor effect that limits long-term discrimination between techniques and may not capture residual disability. Finally, retrospective database research remains susceptible to recording and classification error despite record-level cross-checking.

### 4.2. Clinical Implications

For fractures in which both techniques are technically reasonable, the present results suggest that IMN may be considered when soft-tissue preservation and faster early symptom/occupational recovery are priorities. Plate fixation remains valuable when direct control of the distal fragment, partial articular reduction, or specific alignment goals favor plating. The basis for individualized selection is not a finding that one implant was superior overall. Rather, the plate group had substantially more associated fibular injury/fixation and a longer admission-to-fixation interval, indicating that implant choice tracked clinically meaningful fracture and soft-tissue characteristics that were only partly measured. When these selection differences are considered together with an adjusted early-recovery association but no definitive structural-outcome superiority, the appropriate interpretation is to match fixation strategy to fracture morphology, soft-tissue condition, distal-segment geometry, associated fibular injury, patient goals, and surgeon expertise rather than apply a universal implant preference.

## 5. Conclusions

In this single-center retrospective cohort of adults with AO/OTA 43A-B distal tibial fractures, IMN was associated with less early postoperative pain, earlier documented return to work, and a modestly higher 3-month AOFAS score than plate fixation after adjustment. Pain differences converged by 6 months and remained minimal at 1 and 2 years. Delayed union, nonunion, fixation failure, and reoperation were uncommon and did not differ statistically, but the study was not powered to establish structural equivalence. Because treatment groups differed in clinically important fracture-related characteristics that were incompletely measured, these findings should be interpreted as early-recovery associations within selected treatment pathways, not as evidence of causal or universal superiority of IMN.

## Figures and Tables

**Table 1:** Baseline characteristics of the study cohort.

Characteristic	IMN (*n* = 35)	Plate Fixation (*n* = 52)	*p* Value	SMD
Age, years	46.6 ± 14.3	47.2 ± 15.8	0.847	−0.04
Male sex	25 (71.4%)	27 (51.9%)	0.079	0.40
BMI, kg/m^2^	23.07 ± 2.70	24.55 ± 3.12	0.021	−0.51
Right-sided fracture	16 (45.7%)	18 (34.6%)	0.372	0.23
AO/OTA type B fracture	5 (14.3%)	11 (21.2%)	0.574	−0.18
Current smoking	12 (34.3%)	19 (36.5%)	1.000	−0.05
Alcohol use	14 (40.0%)	19 (36.5%)	0.823	0.07
Diabetes mellitus	8 (22.9%)	12 (23.1%)	1.000	−0.01
Hypertension	10 (28.6%)	22 (42.3%)	0.258	−0.29
Preoperative VAS	7.29 ± 0.71	7.48 ± 0.73	0.217	−0.27
Associated fibular fracture	25 (71.4%)	48 (92.3%)	0.016	−0.56
Fibular internal fixation	18 (51.4%)	44 (84.6%)	0.001	−0.75

Values are mean ± standard deviation or number (percentage). IMN, intramedullary nailing; SMD, standardized mean difference; BMI, body mass index; VAS, visual analog scale.

**Table 2:** Unadjusted clinical outcomes.

Outcome	IMN (*n* = 35)	Plate Fixation (*n* = 52)	*p* Value
Admission-to-fixation interval, days	3.66 ± 1.00	6.10 ± 3.00	<0.001
Postoperative hospital stay, days	3.71 ± 1.07	4.25 ± 1.22	0.034
Operative time, min	83.3 ± 14.1	90.8 ± 27.0	0.093
Intraoperative blood loss, mL	54.6 ± 20.5	69.2 ± 25.8	0.004
Return-to-work time, days	64.6 ± 16.3	98.9 ± 33.6	<0.001
AOFAS score at 3 months	91.34 ± 3.75	88.52 ± 4.86	0.003
VAS, postoperative day 1	5.11 ± 0.93	7.33 ± 1.23	<0.001
VAS, postoperative week 1	3.69 ± 0.99	5.37 ± 0.97	<0.001
VAS, postoperative month 1	1.66 ± 0.73	3.90 ± 1.07	<0.001
VAS, postoperative month 6	0.14 ± 0.36	0.12 ± 0.58	0.786
VAS, postoperative year 1	0.00 ± 0.00	0.06 ± 0.31	0.182
VAS, postoperative year 2	0.00 ± 0.00	0.04 ± 0.28	0.322
Perioperative DVT	10 (28.6%)	12 (23.1%)	0.620
Postoperative infection	0 (0.0%)	3 (5.8%)	0.270
Delayed union	1 (2.9%)	3 (5.8%)	0.646
Nonunion	0 (0.0%)	1 (1.9%)	1.000
Internal-fixation failure	0 (0.0%)	3 (5.8%)	0.270
Reoperation	0 (0.0%)	4 (7.7%)	0.145

Continuous outcomes are mean ± standard deviation; categorical outcomes are number (percentage). *p* values for sparse structural events are from Fisher’s exact test. AOFAS, American Orthopaedic Foot and Ankle Society; DVT, deep-vein thrombosis; VAS, visual analog scale.

**Table 3:** Adjusted and overlap-weighted associations of IMN relative to plate fixation.

Outcome	Adjusted Mean Difference	95% CI	*p* Value	Overlap-Weighted Mean Difference	95% CI	*p* Value
AOFAS score at 3 months	2.53	0.48 to 4.57	0.015	2.70	0.52 to 4.87	0.015
Admission-to-fixation interval	−1.94	−2.86 to −1.02	<0.001	−1.84	−2.79 to −0.88	<0.001
Postoperative hospital stay	−0.34	−0.89 to 0.21	0.228	−0.29	−0.86 to 0.29	0.326
Operative time	−5.33	−13.29 to 2.62	0.189	−5.79	−15.44 to 3.85	0.239
Intraoperative blood loss	−11.67	−23.58 to 0.25	0.055	−11.04	−22.67 to 0.59	0.063
Return-to-work time	−30.76	−41.53 to −19.99	<0.001	−30.64	−44.03 to −17.26	<0.001

Negative values favor IMN for time and blood-loss outcomes; positive values favor IMN for AOFAS score. Multivariable models adjusted for age, sex, BMI, fracture type, smoking, alcohol use, diabetes, and hypertension. Overlap weighting used age, sex, BMI, fracture side, fracture type, smoking, alcohol use, diabetes, and hypertension. A separate fibular sensitivity analysis is reported in Supplementary Table S1.

**Table 4:** Adjusted early postoperative pain differences from the generalized estimating equation model.

Time Point	Adjusted Mean Difference	95% CI	Holm-Adjusted *p* Value	Interpretation
Day 1	−1.85	−2.34 to −1.35	<0.001	Lower VAS after IMN
Week 1	−1.31	−1.79 to −0.83	<0.001	Lower VAS after IMN
Month 1	−1.88	−2.32 to −1.44	<0.001	Lower VAS after IMN

The treatment-by-time interaction was *p* < 0.001 (Wald test). The model adjusted for baseline VAS, age, sex, BMI, fracture type, smoking, diabetes, and hypertension. Time-specific *p* values were Holm-adjusted. Sensitivity analysis additionally controlling for associated fibular fracture and fibular fixation produced similar estimates (Supplementary Table S1).

## Data Availability

The de-identified data supporting the findings of this study are available from the corresponding author upon reasonable request. Public release of individual-level data is restricted by institutional ethical and privacy requirements.

## Supplementary Materials

The following supporting information can be downloaded at: https://www.mdpi.com/article/10.3390/jcm15176752/s1, Table S1: Sensitivity analyses accounting for associated fibular fracture and fibular fixation.

## Abbreviations

AOFAS, American Orthopaedic Foot and Ankle Society; BMI, body mass index; CI, confidence interval; DVT, deep-vein thrombosis; GEE, generalized estimating equation; IMN, intramedullary nailing; SMD, standardized mean difference; VAS, visual analog scale.
