# Supplementary material for: Intramedullary Nailing Versus Plate Fixation for AO/OTA Types 43A and 43B Distal Tibial Fractures: Early Pain Trajectories, 3-Month Ankle Function, and Return to Work in a Retrospective Cohort
Source: J Clin Med. 2026 Aug 31;15(17):6752. doi: 10.3390/jcm15176752 (PMC13566837; doi:10.3390/jcm15176752)
Supplement: Supplementary file 1 [file jcm-15-06752-s001.zip › jcm-4475771-supplementary.pdf]

Supplementary Materials

Supplementary Table S1. Sensitivity analyses accounting for associated fibular fracture and fibular fixation

Outcome	Primary adjusted model	95% CI	p value	Fibular fracture/fixation-adjusted model	95% CI	p value
AOFAS score at 3 months, points	+2.53	0.48 to 4.57	0.015	+2.06	0.04 to 4.08	0.046
Return-to-work time, days	−30.76	−41.53 to −19.99	<0.001	−29.97	−40.77 to −19.18	<0.001
VAS, postoperative day 1, points	−1.85	−2.34 to −1.35	<0.001	−1.81	−2.30 to −1.33	<0.001
VAS, postoperative week 1, points	−1.31	−1.79 to −0.83	<0.001	−1.28	−1.77 to −0.80	<0.001
VAS, postoperative month 1, points	−1.88	−2.32 to −1.44	<0.001	−1.85	−2.30 to −1.40	<0.001

Values are adjusted between-group differences for intramedullary nailing (IMN) versus plate fixation. Positive values for the AOFAS score favor IMN; negative values for return-to-work time and VAS indicate earlier return to work and lower pain after IMN, respectively. The primary adjusted models used the prespecified covariates in the main analysis. The sensitivity models additionally included associated fibular fracture and fibular fixation. AOFAS, American Orthopaedic Foot & Ankle Society; CI, confidence interval; VAS, visual analog scale.
